# The impact of methamphetamine on liver injury in Iraqi male addicts

**DOI:** 10.1016/j.toxrep.2024.101806

**Published:** 2024-11-08

**Authors:** Nawar S. Mohammed, Zahraa Q. Ali, Aseel Sameer Mohamed, Sazan Abdulwahab Mirza

**Affiliations:** a Department of Biochemistry, College of Medicine, University of Baghdad, Baghdad, Iraq; b Department of Anatomy, College of Medicine, University of Baghdad, Baghdad, Iraq; c Al-Kindy Medical College, University of Baghdad, Iraq; d Department of Pathology, College of Medicine, University of Baghdad, Baghdad, Iraq

**Keywords:** Albumin, GGT, AST, ALT, ALP, Histopathological analysis, Intrahepatic cholestasis

## Abstract

Methamphetamine (METH) is a powerful stimulant that affects neurochemical processes controlling heart rate, appetite, blood pressure, body temperature, and wakefulness, making it highly susceptible to abuse. Liver enzymes such as ALT, AST, ALP, and GGT are crucial for liver function. Albumin, a protein synthesized by healthy liver cells, serves as an indicator of chronic liver disease. Additionally, hepatocytes produce bile acids, which are essential for the secretion of bile salts into the bile canaliculi. Disruption in this secretion results in the accumulation of bile salts in the canaliculi, leading to intrahepatic cholestasis. METH-induced liver toxicity involves disruptions in hepatic metabolism, oxidative stress, and increased body temperature, affecting cellular processes such as cell division and the cell cycle and potentially accelerating liver cell apoptosis. The study explores the link between liver toxicity and hepatocyte damage in Iraqi males suffering from addiction. This is a case-control study, conducted at Ibn-Rushed Psychiatric Hospital in Baghdad from July 2023 to February 2024, involved 196 males, with addiction durations exceeding 24 months with varying degrees of methamphetamine (METH) addiction. The study included 187 healthy male controls with no history of drug addiction. Participants were aged 18–40 years. Diagnosis was confirmed using a drug test screening card administered by a specialist. The study included liver function tests (ALT, AST, ALP, and GGT), total bilirubin, and albumin concentration assessments. Significant differences were observed between the addicts and controls, particularly a marked decrease in serum albumin concentration in the addicted males. The ROC curve classification model at various thresholds demonstrated that liver enzymes, especially ALT, ALP, and GGT, exhibited increased sensitivity to METH addiction. A histopathological examination was conducted on a deceased 38-year-old male who had a six-year history of chronic amphetamine addiction to confirm liver injury and the resulting elevation of liver enzymes. The findings of this study indicate that METH greatly affects liver function, suggested to the importance of following a preventative measures and effective treatment approaches for monitoring METH addiction progress.

## 1 Introduction

The number of individuals addicted to methamphetamine (METH) in Iraq is increasing, which poses significant risks to both health and society. METH is a widely known stimulant that impacts various neurochemical functions, affecting blood pressure, temperature, appetite, heart rate, alertness, and stress response. Methamphetamine exists in two main forms: crystal meth and pure D-methamphetamine [1], [2]. Differentiating between these forms is essential for understanding the distinct pharmacological effects and potential therapeutic uses of the drug. The N-methyl derivative is prescribed for medical conditions such as obesity, narcolepsy, attention-deficit/hyperactivity disorder (ADHD), and various sleep disorders. However, the misuse of methamphetamine is widespread and can profoundly disrupt normal cellular and molecular functions [2].

Methamphetamine use is associated with numerous adverse effects, including the generation of reactive oxygen species (ROS), oxidative stress, accelerated aging, apoptosis, and necrosis [2]. It can be ingested orally, intravenously, or through a combination of both methods, rapidly entering the bloodstream and spreading throughout the body, with major organs like the lungs, brain, liver, and kidneys absorbing significant amounts [1], [2], [3].

Methamphetamine exerts a strong stimulant effect on the central nervous system (CNS), resulting in pronounced euphoria, increased alertness, and reduced appetite [4]. The primary mechanism of action involves the release of dopamine and serotonin within the CNS [5]. Research using animal models indicates that methamphetamine can provoke inflammation and oxidative stress, which may contribute to respiratory, cardiovascular, and neurological complications [6], [7]. Oxidative stress refers to an imbalance between oxidants and antioxidants, causing damage to essential macromolecules when free radicals accumulate. In research on methamphetamine's oxidative effects on dopamine in rats, it was observed that reactive oxygen intermediates increased in synaptosomes, although this effect was absent in animals without dopamine synaptosomes [8], [9], [10].

METH is associated with hepatic inflammation and various behavioral and neurotoxic outcomes including neuroinflammation, oxidative stress, autophagy, and neuronal demise. The widespread accessibility and detrimental repercussions of METH have contributed to heightened health concerns and mortality rates [11], [12], [13], [14]. METH induces hepatotoxicity through intricate pathways, including the disruption of liver metabolic processes, the generation of oxidative stress, and the elevation of body temperature. It has been associated with liver damage, potentially accelerating hepatic apoptosis by disrupting essential biological functions such as cell division and the cell cycle [15]. Additionally, a key feature of pathophysiology is the accumulation of bile salts, especially within the bile canaliculi. It has been proposed that lower levels or compromised function of hepatocytes result in decreased stability of the plasma membrane [16].

METH induces pronounced hyperthermia, which is associated with neuronal damage through disturbances in body temperature regulation. The notable hyperthermia (39–42°C) observed following METH exposure is likely a consequence of elevated monoamine levels induced by the drug. This leads to a complex interplay involving hyperlocomotion, metabolic alterations, changes in hypothalamic neurotransmission, and vasoconstriction. Additionally, hyperthermia, similar to that seen in heat stroke, induces hepatocellular morphological changes resembling those observed 24 hours after acute METH exposure [17].

Alanine aminotransferase (ALT), an enzyme presents in hepatocytes, and aspartate aminotransferase (AST), an enzyme primarily found in multiple tissues, with the liver having the highest concentration, it is also found in the heart, brain, kidney, red blood cells, and skeletal muscle. Elevated levels of ALT and AST in the bloodstream indicate the presence of liver damage [18]. Alkaline phosphatase (ALP) is a group of enzymes that catalyze the hydrolysis of several organic phosphate esters at an alkaline pH. The liver (in the biliary tree), bone, intestine, and placenta in pregnant women contains the majority of ALP. Elevated ALP levels are observed in conditions such as cholestasis and bone metastases, where damage to the canalicular membrane occurs [19]. Furthermore, the gamma-glutamyl transferase enzyme (GGT) facilitates the transfer of the gamma-glutamyl group among peptides. Its primary location is in the liver; however, GGT is also present in other organs, including the prostate, kidneys, pancreas, intestine, and spleen. Elevated serum GGT levels serve as an indicator of biliary epithelium injury, while elevated levels of ALT and GGT together indicate cholestasis. [20]. Moreover, bilirubin, a yellow compound produced as a byproduct of the normal breakdown of red blood cells, is processed by the liver, where it is conjugated and subsequently excreted with bile. In cases of liver injury, the liver's ability to process and excrete bilirubin can be impaired. If the hepatocytes are damaged, they may not effectively conjugate bilirubin, leading to an accumulation of unconjugated bilirubin in the blood [21]. Additionally, the excretion of conjugated bilirubin into bile can be hindered; this leads to an increase in bilirubin levels in the blood. Furthermore, failure in the excretion of bilirubin may result in jaundice, anemia and pruritus due to the accumulation of bilirubin in the skin. Therefore, total bilirubin is utilized as a key marker for assessing liver function and diagnosing liver injury [22]. Moreover, albumin, which is the primary protein in the bloodstream, is primarily synthesized by healthy liver cells. Decreased levels of albumin have been identified in the serum of individuals experiencing hepatocellular damage [11]. Bile production and flow are essential for maintaining health. Hepatocytes synthesize bile acids, primarily cholic and chenodeoxycholic acids, which are conjugated with taurine or glycine to form more water-soluble bile salts. These salts are secreted into the duodenum, where a portion is reabsorbed into the portal circulation, forming the enterohepatic circulation [23], [24]. The release of bile salts into the bile canaliculi, small spaces between hepatocytes, depends on proper hepatocyte function.

Renal excretion and hepatic metabolism are the two pathways through which methamphetamine leaves the body [25]. The primary site of metabolism is the liver, which generates metabolites as 4-hydroxyamphetamine, 4-hydroxynorephedrine, amphetamine, norepinephrine, and 4-hydroxymethamphetamine [26]. Methamphetamine overdose and repeated ingestion can severely impair several organ systems, leading to acute renal failure, pulmonary toxicity, neurotoxicity, and cardiotoxicity. Elevated doses of methamphetamine can activate the cardiovascular system, leading to increased blood pressure and a possibly life-threatening rise in heart rate [27].

This research intends to examine and clarify the possible relationships between liver toxicity and hepatocyte damage in Iraqi males suffering from methamphetamine (METH) addiction. By analyzing the biochemical and pathological alterations occurring in the liver, the study aims to enhance our understanding of the effects of METH addiction on liver function and its role in hepatic injury among this demographic.

## 2 Materials and methods

The present study is a case-control one that conducted at Ibn Rushed Psychiatric Hospital in Baghdad. The study included 196 males diagnosed with varying degrees of methamphetamine (METH) addiction, with addiction durations exceeding 24 months. Additionally, a control group of 187 healthy males with no history of methamphetamine use or addiction to nicotine, caffeine, or any other substances was included. The control group had no previous medical conditions such as liver disease, kidney disease, ischemic heart disease, or diabetes mellitus. The data collection spanned from February to July 2022. The range of participants age was from 18–40 years. A drug test screening card, administered by a specialist counselor, was utilized for diagnosis. Additionally, a specialized counselor utilized a drug test screening card to diagnose substance abusers. A positive result was indicated by the absence of a test line, marked as "T," on the amphetamine test strip.

### 2.1 Exclusion criteria

The study exclusively targeted male participants, with no female involvement. Individuals addicted to alcohol, nicotine and caffeine. Also, other substances (e.g. Paracetamol, Statins, anti-tubercular drugs, chemotherapy, herbal medicines), or drugs were intentionally excluded from the study. Furthermore, participants with a medical history of liver diseases (e.g. Viral Hepatitis (Hepatitis B, Hepatitis C)), kidney diseases, diabetes mellitus, heart failure, ischemic heart diseases, or hypertension were also excluded from the study.

### 2.2 Biochemical analysis

Blood samples without EDTA were taken and allowed to sit at room temperature for one hour. They were then centrifuged at 5000 ×g for 20 minutes. The serum obtained was collected and stored for liver function tests, which were performed using the Beckman Coulter 480 AU Chemistry Analyzer, a diagnostic device from Beckman Coulter, Germany. The tests measured the levels of ALT, AST, GGT, ALP, and albumin.

### 2.3 Statistical analysis

Using SPSS version 26, the current data were statistically analyzed. The data was presented using descriptive statistics, including bar charts and the Mean with Standard Deviation. One sample t-test was used to evaluate the correlation between continuous variables. The sensitivity and specificity of the tests were evaluated, and the cutoff value was established using Receiver Operating Characteristic (ROC) curve analysis. A degree of confidence in study findings is referred to as statistical significance, and it is commonly expressed by the P-value. When the P-value was equal to or less than 0.05, statistical significance was taken into account in this investigation.

## 3 Results

**Fig. 1:**
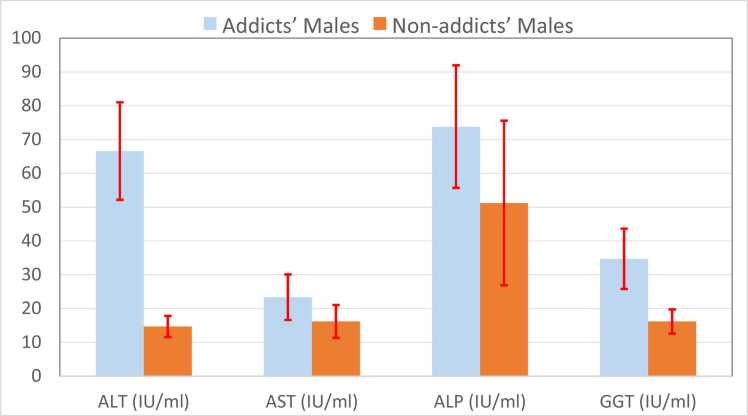
The mean ±SD of liver enzymes, total bilirubin, and albumin for addict and non-addict males.

The present work results showed that there were significant differences in mean ± SD of the liver enzyme levels; ALT, AST, ALP, and GGT between the addicts’ males and non-addicts’ males. Also, the same was found for albumin concentration and serum urea in the blood, as listed in Table 1. Fig. 1 presents the mean ± SD of liver enzyme levels in the blood for both non-addict and addict males. These values illustrate the average levels and variability of these biomarkers in each group, highlighting the differences between those without addiction and those with METH addiction.

**Table 1:** The differences in mean ±SD between the addicts' males and non-addicts’ males.

Variables	Mean ± SD (Addicts’ men) N= 196	Mean ± SD (non-addicts’ men) N= 187	P- value
Age	27.28 ± 6.78	29.34 ± 7.14	0.631
BMI (kg/m^2^)	19.61 ± 3.58	21.78 ± 5.62	0.274
ALT (IU/mL)	66.59 ± 14.43	14.70 ± 3.12	0.001**
AST (IU/mL)	23.35 ± 6.75	16.18 ± 4.85	0.023*
ALP (IU/mL)	73.82 ± 18.14	51.23 ± 24.35	0.002**
GGT (IU/mL)	34.71 ± 8.92	16.15 ± 3.57	0.030*
Total bilirubin (mg/dL)	1.12 ± 0.23	0.86 ± 0. 21	0.431
Albumin (g/mL)	27.37 ± 4.32	34.10 ± 7.64	0.048*
Urea (mg/mL)	8.76 ± 2.34	3.52 ± 0.74	0.020*
Creatinine (µmol/L)	81.65 ± 19.21	76.49 ± 16.45	0.625

* Statically significant

** Statically highly significant.

The sensitivity and specificity of the tests were evaluated, and the cutoff value was established using Receiver Operating Characteristic (ROC) curve analysis, the standard ROC curve test quality is listed in Table 2.

**Table 2:** Standard ROC curve.

Area Under Curve Value	Test Quality
0.9–1.0	Excellent
0.8–0.9	Very good
0.7–0.8	Good
0.6–0.7	Satisfactory
0.5–0.6	Unsatisfactory

The ROC curves results of the liver enzymes, total bilirubin, albumin, urea, and creatinine are listed in Table 3.

**Table 3:** ROC curves results.

**Variable**	**Area Under Curve Values**	**P –Value**	**Cut off**	**Asymptotic 95 % Confidence Interval**		**Test Quality**
				**Lower**	**Upper**	
**ALT**	0.917	0.000**	68.54	0.851	0.983	Excellent
**AST**	0.771	0.000**	23.50	0.664	0.879	Good
**ALP**	0.999	0.000**	43.32	0.997	1	Excellent
**GGT**	0.816	0.000**	18.98	0.720	0.912	Very good
**Total Bilirubin**	0.564	0.349	61.44	0.434	0.695	Fair
**Albumin**	0.647	0.033*	15.05	0.513	0.780	Satisfactory
**Urea**	0.762	0.000**	3.90	0.651	0.874	Good
**Creatinine**	0.666	0.016*	14.20	0.542	0.789	Satisfactory

### 3.1 ROC curve analysis

**Fig. 2:**
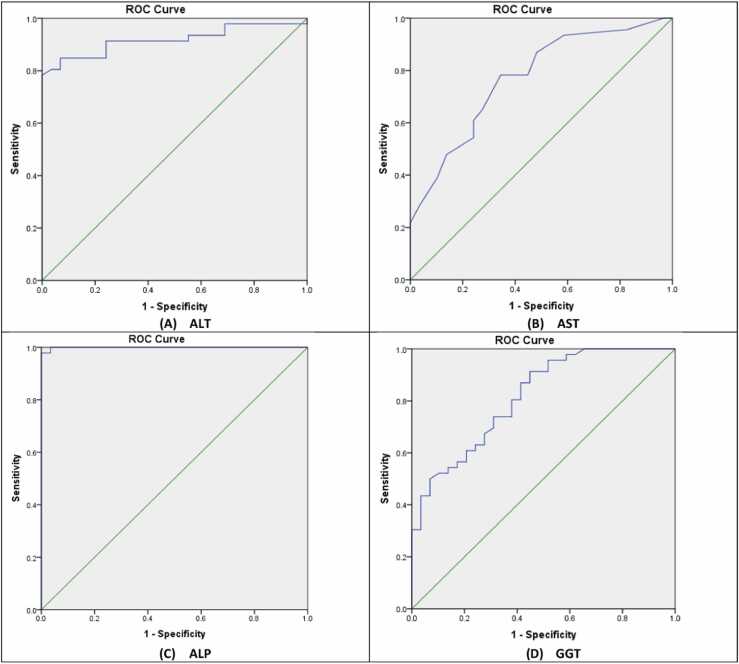
ROC curves of liver enzymes, ALT, AST, ALP, and GGT.

1. ALT: An AUC of 0.917 is considered excellent and indicates that ALT is highly effective in distinguishing individuals with liver injury from those without. This strong performance highlights ALT as a reliable biomarker for detecting liver dysfunction, supporting its well-established clinical role in assessing liver health. The cut-off value of 68.54 for ALT represents the threshold that best balances sensitivity and specificity in current analysis. In clinical terms, this means that ALT levels above 68.54 are highly indicative of liver injury or dysfunction. The p-value of 0.000 indicates that the results are statistically significant, Fig. 2-A.

**Fig. 3:**
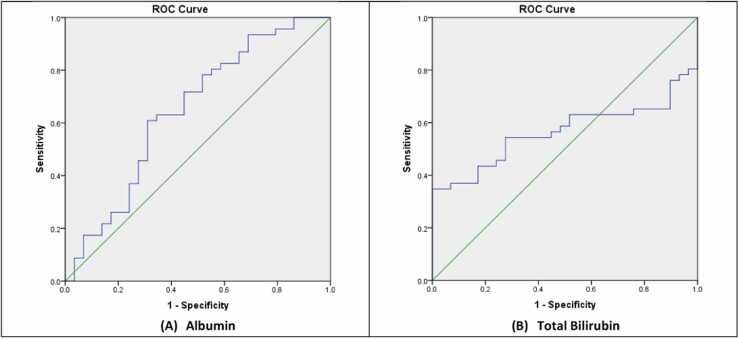
ROC curves of the albumin and total bilirubin concentrations.
2. ALP: An AUC of 0.999 is exceptionally high this implies that ALP is an almost flawless biomarker for distinguishing between individuals with and without liver injury or dysfunction in the context of this study. Such an impressive AUC value indicates that ALP can correctly identify cases with a minimal risk of misclassification, making it extremely reliable for diagnostic purposes. The cut-off value of 43.32 for ALP is the point that best separates individuals with liver dysfunction from those without, balancing sensitivity and specificity. This means that ALP levels exceeding 43.32 are highly indicative of liver issues. A well-defined cut-off like this is crucial for clinical applications, as it helps clinicians make accurate and informed decisions about patient management. The relatively low threshold of 43.32 suggests that even slight elevations in ALP may be significant in detecting liver damage. The p-value of 0.000 indicates that the results are statistically significant, confirming that ALP’s ability to differentiate between cases is extremely unlikely to be due to random chance. This strengthens the validity of the findings and supports the use of ALP as a reliable biomarker for liver function assessment, Fig. 2-C.
3. AST: An AUC of 0.771 indicates a good level of diagnostic accuracy, suggesting that AST is effective in distinguishing between individuals with and without liver dysfunction. While not as high as an AUC value above 0.8 (which would indicate stronger discrimination), an AUC over 0.7 is still considered acceptable and reliable for diagnostic purposes. This result implies that AST can be a useful marker in identifying liver injury, though it may be more effective when used in combination with other diagnostic tests for a comprehensive assessment. The cut-off value of 23.50 for AST denotes the threshold that best balances sensitivity and specificity in this analysis, which is an indication with liver injury or dysfunction. This cut-off provides a practical guide for clinicians, allowing them to interpret AST values effectively. The p-value of 0.000 indicates that the findings are statistically significant, as shown in Fig. 2-B.
4. GGT: GGT was found to have good discriminatory power as the AUC of value 0.816 displays good diagnostic accuracy, which implies that GGT can efficiently identify patients suffering from liver dysfunction and those without. This finding suggests that GGT can be regarded as a good marker for liver injury, further upholding its significance in clinical evaluation of the liver. The cut-off value of 18.98 for GGT optimizes sensitivity and specificity, so there is effective discrimination of the cases. GGT levels above 18.98 ought to be clinically actionable since they are suggestive of liver damage or dysfunction. The authors set this value in order to assist clinicians in interpreting GGT levels, so that all patients with levels above 18.98 receive further attention or intervention for liver damage. The p-value of 0.000 indicates that the results are unequivocally significant showing that the discriminative power of GGT as observed cannot be due to chance. This serves to strengthen the validity of your findings and further explains the usefulness of GGT in liver assessment, Fig. 2-D.
5. Urea: An AUC of 0.762 indicates a moderate level of diagnostic accuracy. While not as strong as an AUC above 0.8, an AUC greater than 0.7 still signifies that urea is reasonably effective in distinguishing between individuals with and without liver injury resulting from METH exposure. This suggests that urea levels could be a meaningful indicator in assessing liver damage in the context of METH toxicity, but the diagnostic performance is moderate. Therefore, urea should likely be used alongside other markers to improve overall diagnostic accuracy. The cut-off value of 3.90 for urea identifies the threshold at which there is an optimal balance between sensitivity and specificity. Clinically, urea levels above 3.90 may indicate liver dysfunction or damage, especially in the context of METH-related toxicity. This threshold can help clinicians assess the severity of liver injury, but it should be noted that urea levels can also be influenced by kidney function, protein intake, and hydration status, which may impact the interpretation. The p-value of 0.000 indicates that the findings are statistically significant, Fig. 4-A.

**Fig. 4:**
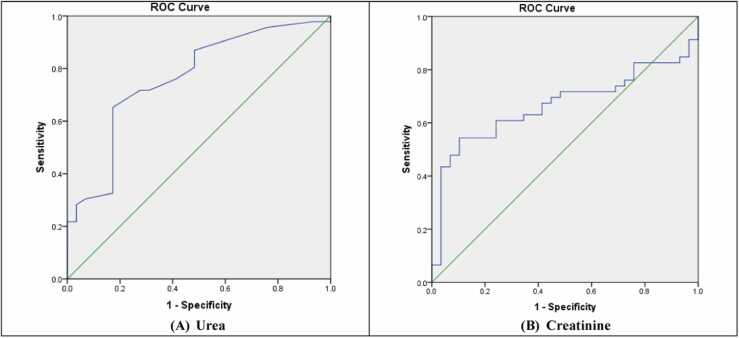
ROC curves of the urea and creatinine concentrations.

### 3.2 Clinical implications of liver enzymes in METH exposure

ROC curve analysis revealed the potency of methamphetamine (METH) in liver enzyme activities, with the sensitivity of ALT, AST, ALP, and GGT much higher. This indicates that hepatotoxic effects from prolonged consumption of METH or a high dose may specifically affect these enzymes. Of the tested enzymes, ALT presents with the highest diagnostic quality evidenced by its substantial AUC value and statistical significance. The established cut-off value gives a concrete boundary which will help the physician to detect liver injury thus managing the patient which informed decisions. The credibility of hepatocellular injury is strong when there is an increase in ALT levels above this threshold.

ALP also emerges as a highly effective biomarker for diagnosing liver dysfunction, supported by its exceptional AUC value and statistical significance. This makes ALP a reliable tool in clinical assessments of liver health, with potential applicability across diverse patient populations. The findings reinforce the importance of ALP as a sensitive indicator of liver injury, meriting further exploration in various clinical scenarios.

AST, while not as definitive as ALT, still provides valuable diagnostic insight with its good discriminatory capacity. The AUC value suggests that AST is a reliable, though complementary, marker for liver dysfunction. Its cut-off value aids in identifying individuals at risk of liver injury. However, clinicians should consider other factors, such as muscle injury or cardiac conditions, which can influence AST levels and affect specificity. Despite these variables, the significant p-value confirms AST’s utility within the context of the study, though it is best used alongside other liver function tests for a more comprehensive evaluation.

GGT shows good diagnostic accuracy, with an AUC value reflecting its reliability in assessing liver dysfunction. Its clinical relevance is supported by a meaningful cut-off value, indicating that moderate increases in GGT warrant further examination of liver health. However, GGT should be interpreted in conjunction with other markers and patient history, as it can also be elevated due to alcohol consumption, medication use, or other liver conditions, such as hepatitis or bile duct obstruction. Its significance in liver health assessment is amplified when used as part of a diagnostic panel.

Furthermore, the analysis of blood urea levels, depicted in Fig. 4, suggests that urea could be a sensitive indicator of METH-related metabolic disturbances, although it is more commonly associated with kidney function. Elevated urea may reflect disrupted protein metabolism and impaired liver processing, outcomes of METH-induced hepatotoxicity. While urea’s diagnostic role in liver injury is less direct, it provides insight into the systemic effects of METH toxicity, emphasizing the need for a holistic approach when interpreting metabolic markers.

Overall, these findings highlight the complex interplay between METH use, metabolic disruption, and liver enzyme elevations. They advocate for the integration of multiple biomarkers in clinical assessments to ensure accurate diagnosis and effective monitoring of liver health in individuals exposed to METH.

### 3.3 Histopathological results for a single deceased case

**Fig. 5:**
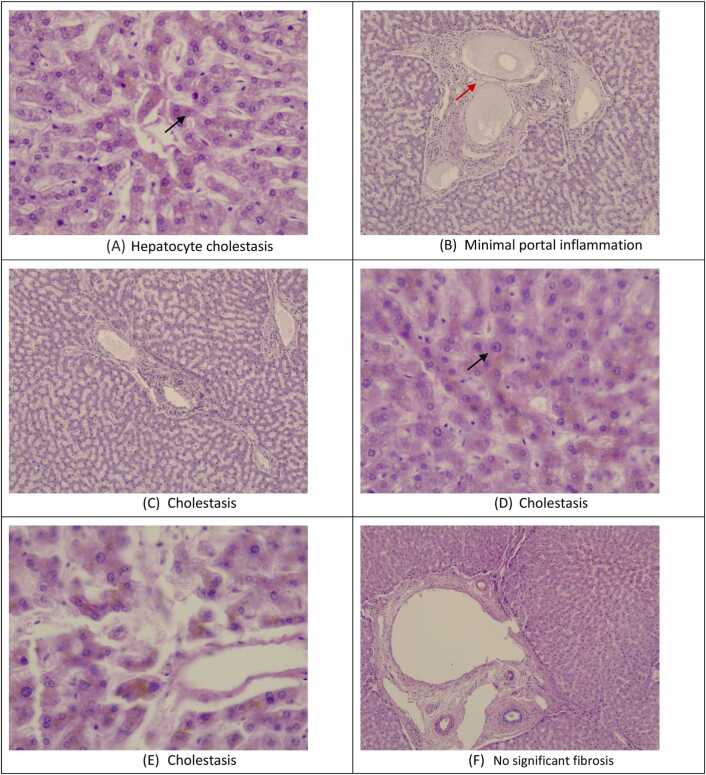
**:** Representative photomicrographs of liver tissues that were stained with hematoxylin–eosin staining, (A) Hepatocyte cholestasis; bile salts accumulation inside hepatocyte, (B) Minimal portal inflammation; mononuclear inflammatory cells (lymphocyte), (C), (D), and (E) Cholestasis, (F) No significant fibrosis.

During the course of this research, we encountered a case involving a 38-year-old male patient with a six-year history of chronic methamphetamine addiction who sadly passed away. A histopathological analysis was conducted solely based on this case, as the patient ultimately succumbed to complications arising from prolonged substance abuse. Tissue samples from liver was collected for histopathological examination, which were fixed in 10 % formalin overnight and then embedded in paraffin, followed by sectioned at 4 µm thickness. And then the sections were stained with hematoxylin and eosin solution. After that, all the sections were observed for histopathological changes using the light microscope using a Leica DM750 microscope (Leica Microsystems GmbH, Germany), and using a high-quality digital camera mounted on the microscope, photographs were taken digitally. Histopathological investigation showed cholestasis by degeneration of hepatocytes (**black arrows**) and inflammatory cellular infiltrates (**red arrow**) (Fig. 5).

## 4 Discussion

This study characterized METH-induced hepatocellular damage. The liver plays a vital role in the digestive system, and it provides crucial materials for the breakdown of food. Additionally, it plays a significant role in the endocrine system, blood purification, and metabolic regulation. Any impairment to this organ can pose a threat to body health [28], [29]. Notably, aminotransferases are liver enzymes responsible for facilitating the transfer of alpha-amino groups from aspartate or alanine to the alpha-keto group of ketoglutaric acid which is a glucogenic compound. This enzymatic process yields oxaloacetic acid and pyruvic acid, respectively, the basic compounds for the production of energy in the body.

The current study revealed that the METH abusers exhibited significantly higher levels of ALT and AST compared to the non addicts males, with statistical significance observed (P < 0.05). The elevated levels of liver enzymes in the bloodstream serve as indicators of hepatocytes injury. Based on the findings of our study, it was observed that METH administration led to significant liver structural alterations in adult males. The liver, being responsible for metabolizing toxins and drugs present in the bloodstream, is susceptible to various forms of damage and undergoes changes in response to high external toxicity which result in oxidative stress and inflammation due to the formation of ROS [30]. Furthermore, increased ALP levels are associated with liver injury, independent of bone-related issues. In this instance, elevations in GGT levels are commonly observed. On the contrary, when the source of elevated ALP is related to bone pathology, GGT levels have a tendency to remain within normal ranges. But the elevation of the two enzymes together indicates hepatic biliary dysfunction [30].

To assess if the observed alterations in hepatocyte morphology indicate liver damage, we conducted serum measurements of ALT, AST, ALP, and GGT. AST and ALT are hepatocellular enzymes that are released into the bloodstream when liver cells are damaged, and the elevated level of them is providing supporting evidence that METH leads to liver damage. These results align with the research conducted by Sovri et al. [31] METH was demonstrated to cause liver damage through various mechanisms, including oxidative stress, cell cycle arrest and inflammation [32]. Wang et al. [33] showed that the METH drug resulted in noteworthy histopathological changes in the livers of mice and elevated serum levels of AST and ALT, which indicate liver injury. Also, the clinical and diagnostic importance of changes in blood enzyme levels, including AST, ALT, and ALP, has been well established [34]. Numerous animal studies have demonstrated that methamphetamine can stimulate transcription factors involved in signaling pathways and the regulation of inflammatory genes, including tumor necrosis factor (TNF-α) [35]. Besides, one of the main functions of the liver is synthesizing albumin, therefore low concentration of albumin in the blood stream indicates hepatocellular dysfunction. Also, the hepatocytes are responsible for the synthesis of bile acids and release of the bile salts into the common bile duct, drug-induced hepatic injury were reported to cause intra-hepatic cholestasis [36]. Hepatic cholestasis is a clinical condition that results from a reduction of bile flow and destruction of the normal bile excretory system [37] this condition causing intrahepatic cholestasis due to the accumulation of bile salts and consequently inflammation. Additionally, the liver plays a crucial role in conjugating bilirubin, a byproduct of red blood cell breakdown, for excretion from the body. As a result, individuals who use methamphetamine tend to have lower hemoglobin levels than healthy individuals, which heightens their risk of developing jaundice and anemia [37].

METH-induced hyperthermia has been shown to contribute to liver cell damage, including alterations in hepatocellular morphology and increased levels of AST and ALT, indicating liver dysfunction. Research by Laura E. Halpin et al. [38] demonstrated that preventing hyperthermia in METH-treated rats by cooling their environment reduced liver damage, suggesting that hyperthermia plays a critical role in METH-induced hepatotoxicity. This underscores the importance of hyperthermia as a key factor in both the structural and functional liver damage. The significant hyperthermia (39–42°C) caused by METH exposure is likely due to elevated monoamine levels induced by the drug. This effect is compounded by a complex interplay of factors, including heightened locomotor activity, metabolic alterations, shifts in hypothalamic neurotransmission, and vasoconstriction [38]. Furthermore, hyperthermia plays a vital role in inducing cellular damage and cell death, with the degree of damage being influenced by the intensity and duration of elevated temperatures. The harmful effects of hyperthermia can be attributed to protein misfolding and disruption of the cell cycle [39]. Also, hyperthermia plays a crucial role in cellular damage by inducing oxidative stress. In vitro studies have confirmed that when elevated temperatures are combined with high concentrations of METH, hepatocyte damage happens. Also, all liver enzymes are proteins that can be denatured in elevated body temperature (hyperthermia) and become inactive, which impair body metabolism. Moreover, METH exposure can significantly alter the liver's normal metabolic functions. The drug disrupts pathways involved in lipid, protein, and carbohydrate metabolism, leading to the accumulation of toxic intermediates and metabolic byproducts. For instance, METH can impair the liver's ability to manage glucose and lipid homeostasis, contributing to hepatic steatosis (fatty liver) and energy imbalance. This disruption of metabolic processes strains hepatocytes and may lead to cellular dysfunction, contributing to liver damage [40], [41]. METH, an exceedingly addictive stimulant, has emerged as a significant global public health concern [42], [43].

Methamphetamine enhances the release of neurotransmitters such as norepinephrine, dopamine, and catecholamines at presynaptic nerve terminals. This overstimulation activates postsynaptic receptors and inhibits neurotransmitter reuptake [44], [45]. Elevated neurotransmitter levels can undergo auto-oxidation, generating reactive oxygen species (ROS) and inducing oxidative stress [45]. As a result, the overproduction of ROS and reactive nitrogen species (RNS) overwhelms the liver’s antioxidant defense systems, including enzymes like superoxide dismutase, catalase, and glutathione peroxidase. The resulting oxidative stress leads to cellular damage, oxidizing lipids, proteins, and DNA. This damage destabilizes cellular membranes, impairs mitochondrial function, and activates signaling pathways that exacerbate inflammation and hepatocyte death, further contributing to liver injury [46].

Animal studies have demonstrated methamphetamine’s role in ROS production [42], which can react with macromolecules like proteins, lipids, and DNA, leading to cellular dysfunction through lipid peroxidation. Oxidative stress plays a significant role in damaging biological molecules and is linked to the development of inflammatory diseases [47], [48], [49].

Electron microscopy of the liver following methamphetamine (METH) addiction reveals hydropic changes, intracellular cholestasis, and inflammation. These alterations resemble the generalized hepatocellular damage observed after hyperthermia or heatstroke, which is indicative of membrane damage [50]. The associated cellular injury leads to increased mitochondrial accumulation, suggesting potential mitochondrial dysfunction [51]. Impaired mitochondrial function could significantly impact ammonia metabolism, as key enzymes involved in the urea cycle, such as carbamoyl phosphate synthetase-1 and ornithine transcarbamylase, are located within mitochondria [52]. Elevated ammonia levels can cross the blood-brain barrier, potentially leading to encephalopathy.

To determine whether the observed morphological changes indicate tissue damage, serum levels of AST and ALT were measured. These enzymes are markers of liver cell damage, and their elevation, alongside increased ammonia levels and altered liver cell structure, supports the hypothesis that METH induces liver injury [53]. Although a rise in AST alone could suggest general tissue damage, such as rhabdomyolysis, the concurrent changes in these variables further confirm hepatocellular damage [54]. In addition, other organs potentially affected by METH toxicity include the muscles, heart, and kidneys [55].

Halpin and Yamamoto [56] reported that METH-induced liver damage was associated with increases in brain and peripheral ammonia and elevation in brain dopamine and serotonin content [56]. The elevation of dopamine levels caused by amphetamines triggers heightened oxidative metabolism, resulting in the production of free radicals that can potentially induce cytotoxic effects. Dopamine is implicated in locomotor stimulation, psychosis, and perceptual disturbances, while alterations in norepinephrine levels are linked to alertness, appetite suppression, increased locomotion, and sympathomimetic effects [39], [57]. Methamphetamine is a stimulant that significantly impacts appetite, potentially resulting in weight loss and malnutrition [58]. Therefore, its use can have both direct and indirect effects on public health.

### 4.1 Study limitations

Our study has several notable limitations. Conducting the study in a single center restricts the applicability of our findings to broader populations, and the relatively small sample size may challenge the statistical robustness and precision of our results. Additionally, most patients enrolled had varying severity levels, from moderate to severe and critically ill, making it difficult to interpret the results due to this wide range of conditions. This diversity may affect the generalizability of our findings regarding methamphetamine use. Furthermore, obtaining comprehensive patient data, including information on concurrent medication use and understanding the interactions between methamphetamine and other drugs, was challenging. More research is needed to explore these areas, potentially enhancing health outcomes for individuals with methamphetamine addiction and other medical conditions.

## 5 Conclusion

The study results underscore the diagnostic importance that liver enzymes, including ALT, AST, ALP, and GGT, hold in assessing liver function and detecting hepatocellular injury. Albumin, a healthy liver cell protein, and bilirubin are the two most important diagnostic indicators for this hepatocellular injury. These biomarkers should be considered for the evaluation of METH-induced liver injury to assess the damaging effects of the drug on hepatocyte health. This presents a pressing need for effective prevention strategies and appropriate interventions for individuals suffering from methamphetamine use disorder. Additionally, these findings help to improve the understanding of pathobiological mechanisms associated with METH use, which would help in designing more effective health interventions.

The ROC curve analysis positions ALT as a highly effective biomarker for diagnosing and monitoring liver dysfunction. Its robust performance underscores its clinical utility, although further research is warranted to confirm its applicability across diverse populations. ALP also emerged as an exceptionally accurate biomarker, providing a reliable criterion for assessing liver function. Future investigations should explore its use in various clinical settings to validate its cut-off values more broadly.

GGT exhibited good diagnostic accuracy, reinforcing its significance in evaluating liver health. Future studies should consider potential confounding factors affecting GGT levels to enhance its clinical relevance. Similarly, AST demonstrated moderate effectiveness as a diagnostic marker; while it is valuable, it should be interpreted in conjunction with other biomarkers and clinical data to improve overall diagnostic precision.

Lastly, the analysis of urea indicates moderate utility in detecting liver injury related to METH. However, the interpretation of urea levels should be integrated with additional liver and kidney function tests for a comprehensive assessment. These findings underscore the complex interplay between METH toxicity and metabolic dysfunction, advocating for a multifaceted diagnostic approach in clinical practice.

## Ethics statements

Consent was obtained or waived by all participants in this study. College of Medicine/University of Baghdad issued approval 15–2022. Consent was waived or obtained by all participants in this study. The IRB approval No. 15–2022 was issued from the Ethics Committee of the College of Medicine at the University of Baghdad. The participants received detailed information about the research goals, and data compilation occurred via an extensive questionnaire.

## Author agreement statement

We the undersigned declare that this manuscript is original, has not been published before and is not currently being considered for publication elsewhere. We confirm that the manuscript has been read and approved by all named authors and that there are no other persons who satisfied the criteria for authorship but are not listed. We further confirm that the order of authors listed in the manuscript has been approved by all of us. We understand that the Corresponding Author is the sole contact for the Editorial process. He/she is responsible for communicating with the other authors about progress, submissions of revisions and final approval of proofs.

## Funding

Authors state no funding involved.

## CRediT authorship contribution statement

**Aseel Sameer Mohamed:** Validation, Software, Formal analysis. **Sazan Abdulwahab Mirza:** Validation, Data curation. **Nawar Mohammed:** Writing – review & editing, Writing – original draft, Methodology, Conceptualization. **Zahraa Q. Ali:** Methodology, Data curation.

## Declaration of Competing Interest

The authors declare that they have no known competing financial interests or personal relationships that could have appeared to influence the work reported in this paper.

## Data Availability

Data will be made available on request.
